# A review of the role of anticoagulation for patients with infective endocarditis and embolic stroke

**DOI:** 10.1002/ccr3.556

**Published:** 2016-04-13

**Authors:** Arfah Hazel Preston, Stefan Williams, Judy Archer

**Affiliations:** ^1^ Huddersfield Royal InfirmaryAcre LaneHD3 3EAHuddersfieldUK; ^2^ Pinderfields General HospitalAberford RoadWakefieldWF1 4DGUK; ^3^ Leeds General InfirmaryGreat George StreetLS1 3EXUK

**Keywords:** Anticoagulation, embolic stroke, infective endocarditis, warfarin

## Abstract

Stroke is a common embolic complication of infective endocarditis. The most important treatment to prevent stroke in endocarditis is the initiation of antibiotic therapy. It is unclear whether the initiation of de novo anticoagulation (i.e, warfarin) in patients with infective endocarditis is beneficial, since there are no large or randomized controlled trials in this area. However, this case report suggests, despite the limited evidence, that anticoagulation in this patient caused no harm and could suggest a hint of possible benefit.

## Background

Infective endocarditis has a high mortality, yet there is often a considerable delay between the onset of clinical signs and recognition of the diagnosis 1. Embolic complications can affect many organs, but ischaemic stroke is one of the most common, occurring in 16% of patients in one series 2. We report a patient with infective endocarditis and stroke, because it is important for clinicians to be familiar with this presentation, so that the diagnosis is not delayed. In addition, our patient was treated with anticoagulation. Intuitively, this might be expected to have a benefit by reducing the risk of further embolic stroke, or alternatively it might be expected to have a deleterious effect, by increasing the risk of intracerebral hemorrhage. In this regard, there are no established guidelines for clinicians and research is limited. The case provides the opportunity to summarize the evidence for and against this intervention.

## Case Presentation

A 25‐year‐old female was admitted with sudden onset of right hemiparesis. This had been preceded by 2 weeks of gradual, mild weakness and numbness of the right leg. There was also a longer history of 6 months of fevers, malaise, and 16 kg weight loss. She had emigrated from Poland, and had been given several courses of antibiotics before coming to the UK, for presumed urinary tract infection. In the UK, her GP had made a diagnosis of atypical migraine.

On examination, the patient was pyrexial at 39.8°C. On cardiac auscultation, there was a grade 5 pansystolic murmur with no peripheral stigmata of infective endocarditis. The neurological examination confirmed a dense right hemiparesis (power grade 0), with right facial weakness, and expressive dysphasia. There were brisk deep tendon reflexes on the right, and the right plantar was upgoing.

There were no risk factors for infective endocarditis evident in either the history or examination.

## Investigations

**Figure 1:**
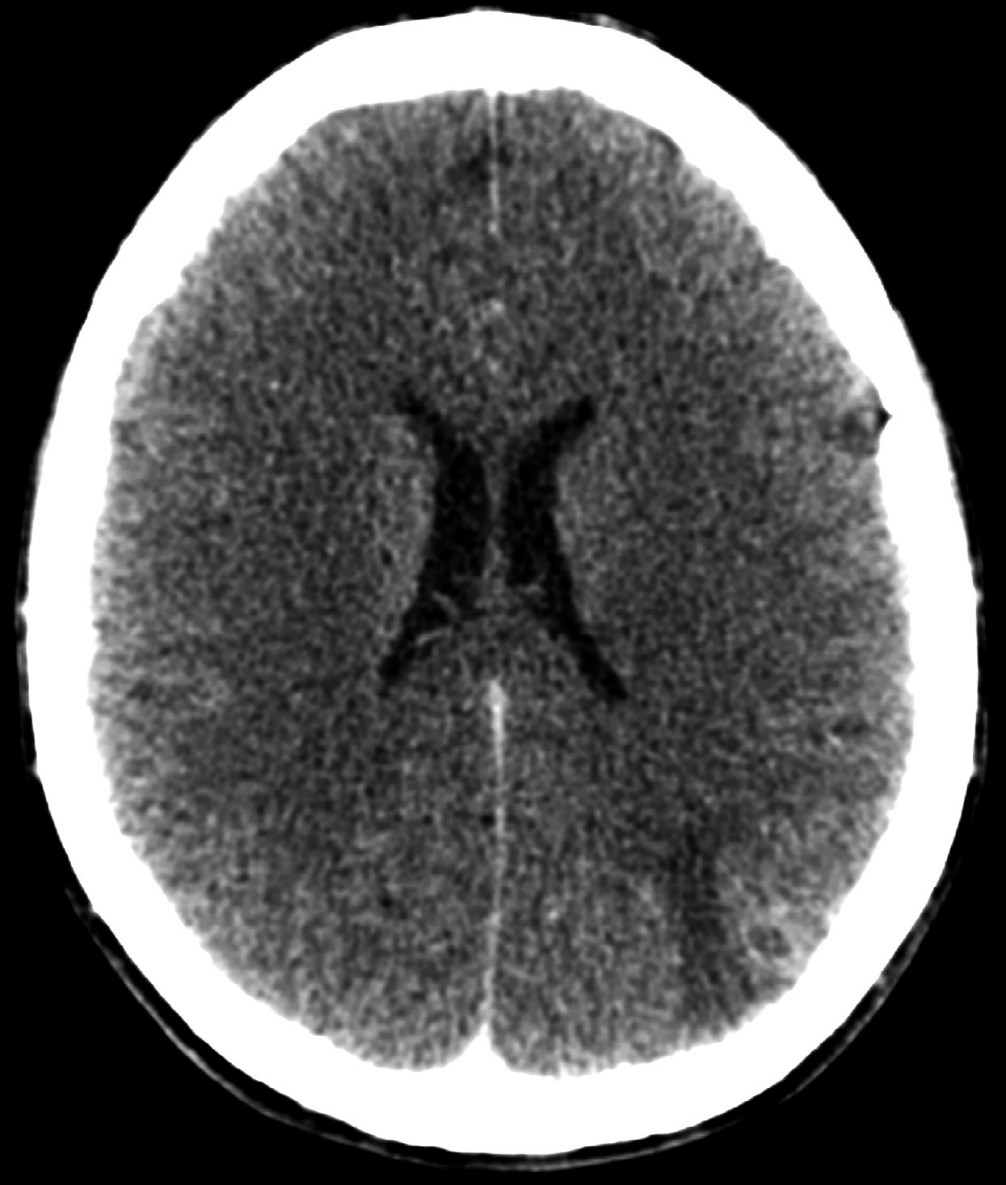
Contrast CT demonstrating hypodensity in Left frontal parietal lobe and Left parietal ring‐enhancing lesion.

**Figure 2:**
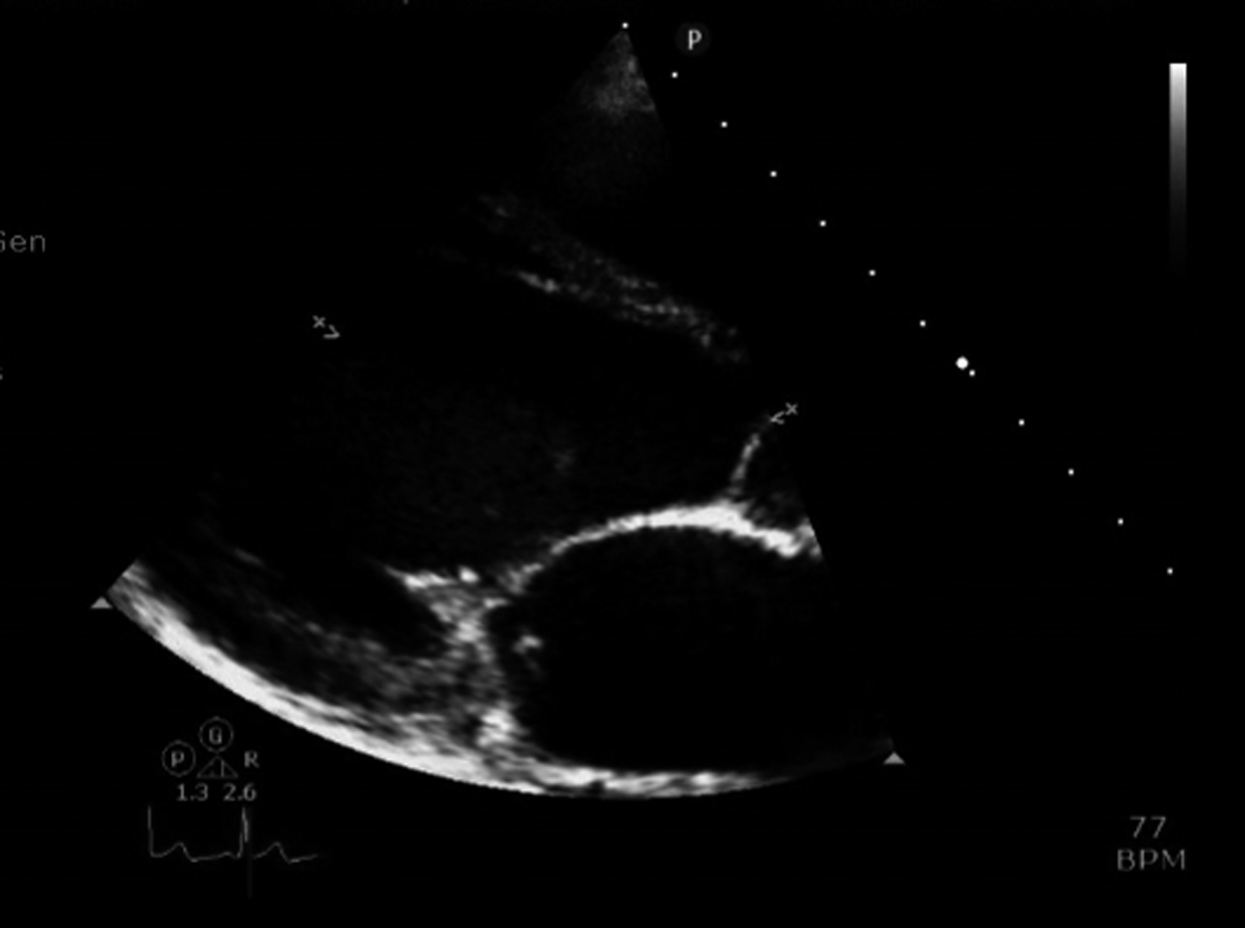
Parasternal view showing thickened mitral valve and vegetation.

Blood tests showed elevated C‐reactive protein (89 mg/L) and neutrophil count (8.69 ×10^9^/L), Table 1. Electrocardiogram showed sinus rhythm. An urgent CT head revealed a left fronto‐parietal hypodensity, and an ill‐defined ring‐enhancing lesion at the left parietal lobe (Fig. 1). On transthoracic echocardiography, there was a 5 mm vegetation on the anterior mitral valve leaflet, which was seen to prolapse (Fig. 2). There was also severe, eccentric mitral regurgitation, and bowing of the intra‐atrial septum.

**Table 1:** Summary of laboratory investigation

**FBC**		**LFT**	
Hb	106 × 10^9^/L	Alb	35 g/L
WCC	10.8 × 10^9^/L	Alt	10 μ/L
MCV	80.7	Alp	94 μ/L
Plts	240	Br	14 μmol/L
Neutrophils	8.69 × 10^9^/L		
Eosinophils	0.03 × 10^9^/L	Glucose	5.8 mmol/L
Lymphocytes	1.00 × 10^9^/L	ESR	25 mg/L
**UE**		**COAG**	
Na	137 mmol/L	PT	10.7 sec
K	3.9 mmol/L	APTT	30.0 sec
Urea	3.0 mmol/L	Fibrinogen	3.4 g/L
Creatinine	53 mmol/L		
CRP	85 mg/L	Urine	Negative
		HIV	Negative

**Figure 3:**
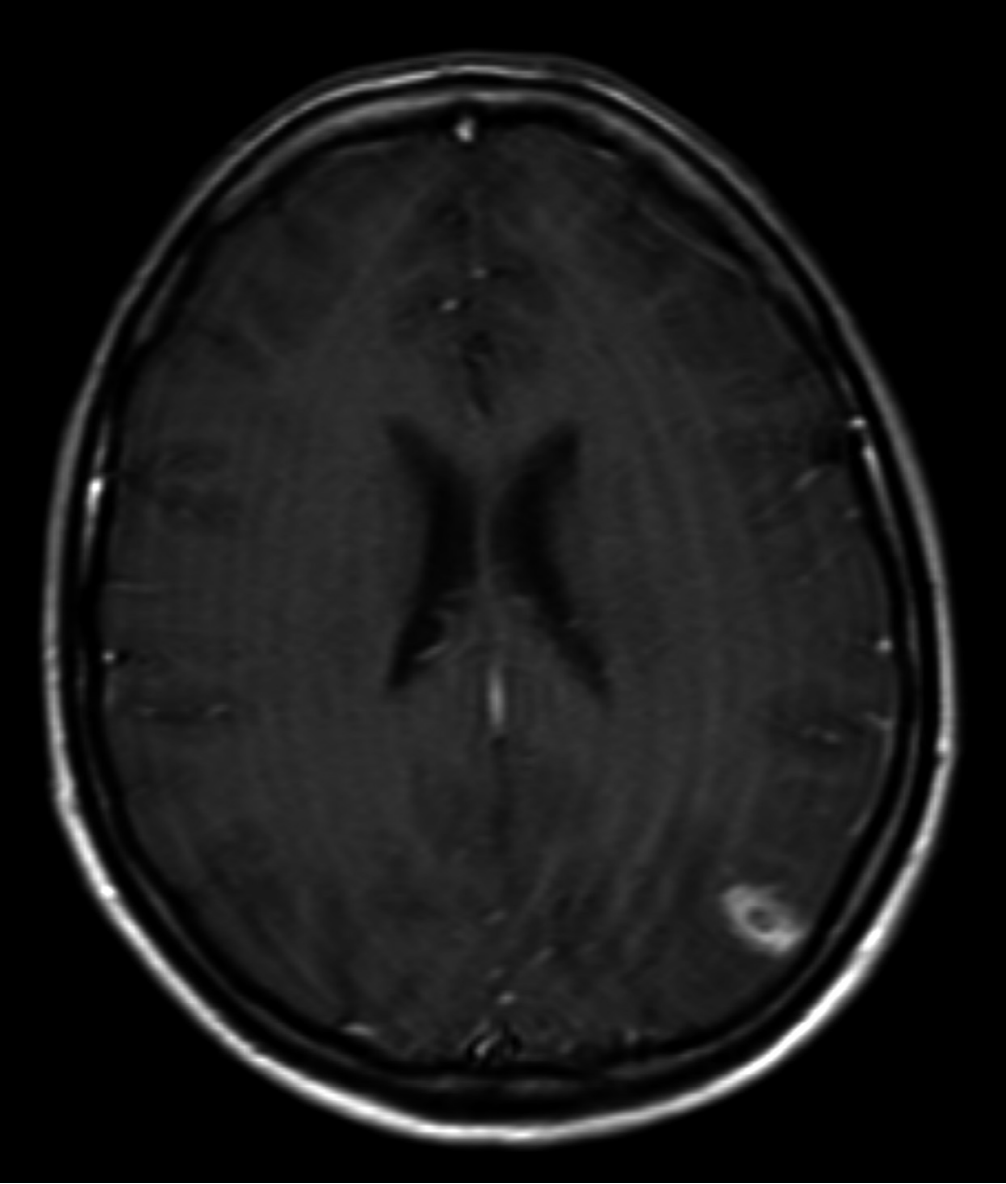
MRI with contrast demonstrating ring‐enhancing lesion in the Left Posterior watershed territory.

**Figure 4:**
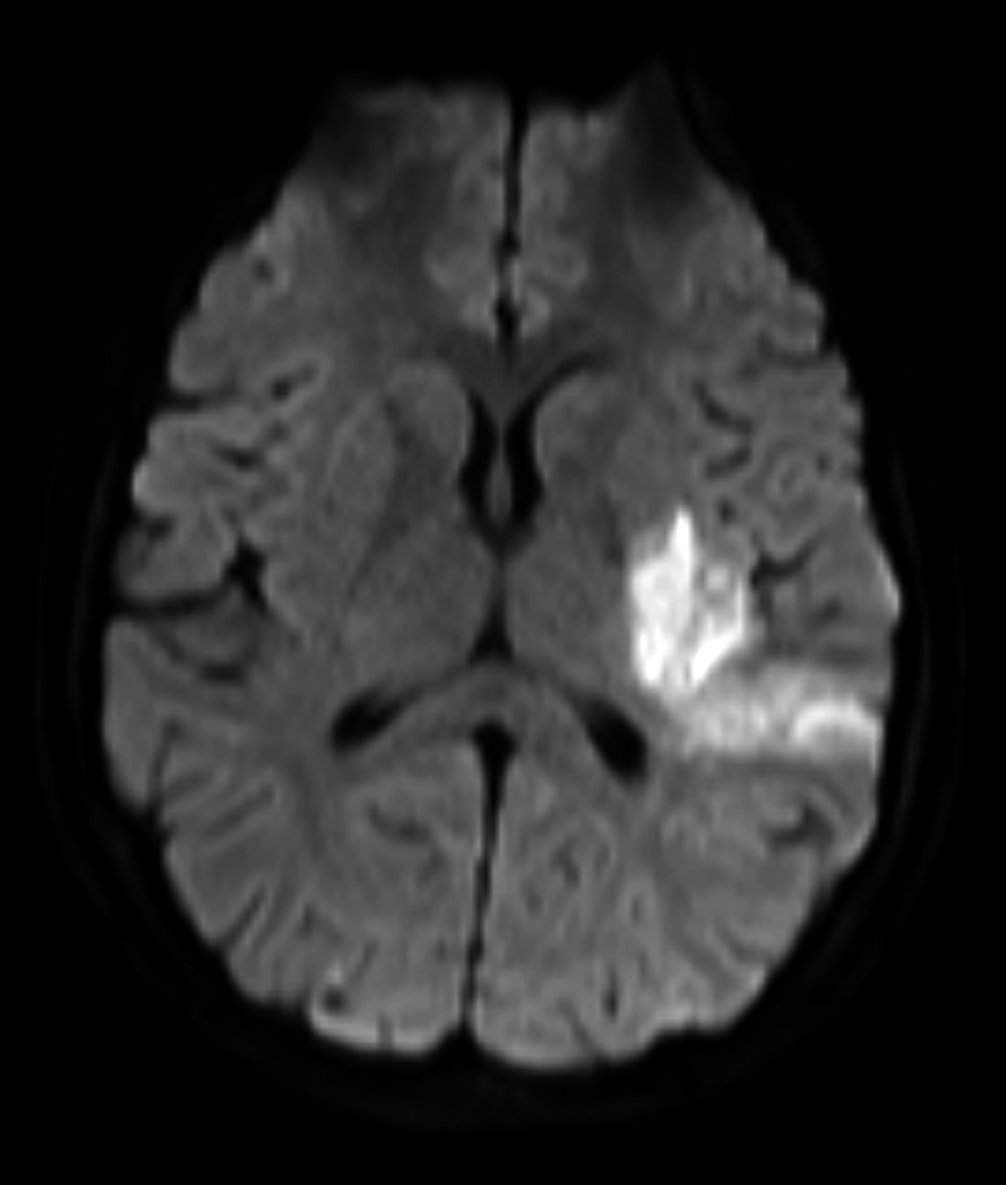
Dense Weight Imaging (DWI) sequence view showing infarction in left territory.

**Figure 5:**
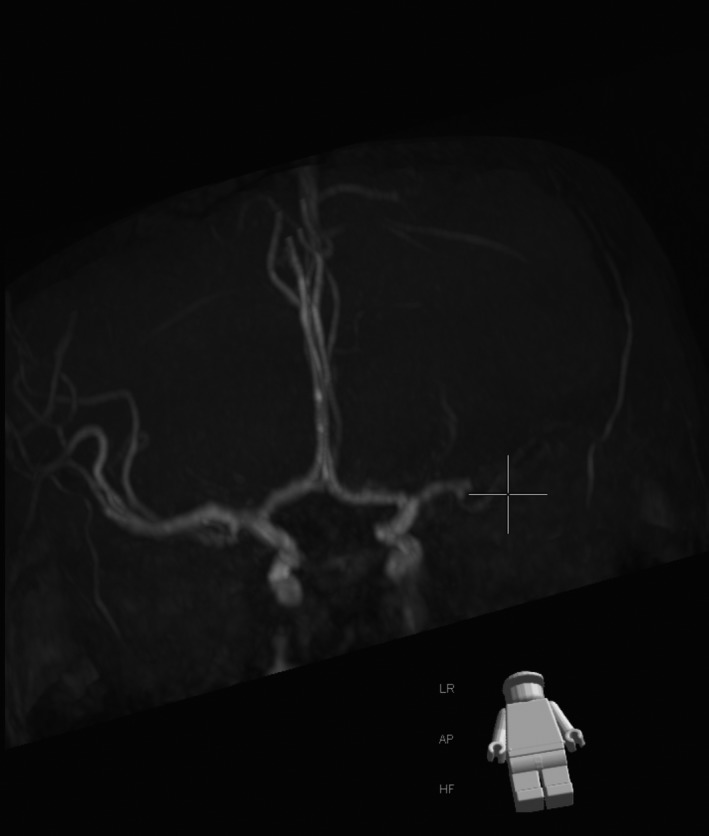
Magnetic Resonance Angiography (MRA) demonstrating left middle cerebral artery (MCA) occlusion.

MRI head (on the day after admission) revealed a ring‐enhancing lesion with surrounding vasogenic edema which was thought to represent an early brain abscess in the left posterior watershed territory (Fig. 3). In addition, diffusion‐weighted imaging sequences confirmed an acute infarction in the territory of the left middle cerebral artery (MCA) (Fig. 4), and MR angiography suggested that the M1 segment of the MCA was occluded (Fig. 5). There were multiple foci of microhemorrhage throughout the brain. CT angiography (CTA) confirmed the occlusion in the left MCA.

A repeat MRI 3 weeks after admission suggested that an intracerebral abscess may have developed within the area of the previous left MCA infarct. However, the patient was clinically improving with antibiotics alone, and a further interval CT showed improved appearances. Interestingly, on the original CTA, the M1 segment appeared slightly bulbous at the site of occlusion, which was thought suspicious for a possible mycotic aneurysm. However, on interval CTA, there was normal morphology of the left M1 segment of the MCA, with no evidence of a mycotic aneurysm.

## Treatment

Joint care was agreed between the stroke and cardiology teams. Empirical antibiotic treatment was started for native valve endocarditis. Subsequently, *Streptococcus mutans* was cultured from three sets of blood cultures. After the initial imaging revealed ischemic stroke and left MCA occlusion, a decision was made by the stroke team to commence anticoagulation with low molecular weight heparin which was subsequently converted to warfarin. She remained anticoagulated for at least 6 weeks with the view to reduce risk of embolic stroke.

There were several discussions with the local cardiothoracic surgery team regarding consideration of valve replacement. However, there were no further embolic events and no signs or symptoms of heart failure. In addition, echocardiography did not show enlargement of the vegetation, and the patient became apyrexial with improving inflammatory markers. As such, elective surgical valve repair was planned.

At the time of discharge to a rehabilitation placement, power had improved to grade 3 in the right arm and leg, and the dysphasia had dramatically improved. She subsequently underwent a successful mitral valve repair with ring angioplasty.

## Outcome and Follow‐up

Routine cardiology and neurology outpatient follow‐up was arranged.

## Discussion

This case is of interest because it involves a decision to initiate anticoagulation in a patient with infective endocarditis and secondary embolic stroke. In this case, she was anticoagulated with Low molecular weight heparin (LMWH) which was subsequently converted to warfarin. Several case series show that the majority of neurological complications occured before the initiation of antibiotic therapy. As such, the most important intervention to prevent further stroke is the initiation of antibiotic therapy 1, 2.

However, there is no established consensus or guideline regarding initiation of de novo anticoagulation in such patients. The decision is intuitively a difficult one. Cardiac vegetations contain blood products such as platelets and fibrin, and so anticoagulation might be expected to reduce the likelihood of embolic stroke. Conversely, infective endocarditis can cause mycotic aneurysm and widespread cerebral microhemorrhage, and so anticoagulation might increase the risk of intracerebral hemorrhage.

There are no randomized controlled trials of anticoagulation in the situation that we describe, but several cohort studies have been reported. A Spanish study looked at *Staphylococcus aureus* endocarditis in patients with prosthetic valves and those with native valves. The majority of the patients with prosthetic valves were taking anticoagulation (warfarin) at the time of admission, and this group showed a much higher rate of intracerebral hemorrhage 4. However, the cases spanned a time period that began prior to the introduction of the Duke criteria for endocarditis, and so there may have been a selection bias toward more unwell patients.

There have been two subsequent cohort studies, involving *S. aureus* and other organisms, with larger numbers of patients and prospective design. Infective endocarditis patients were divided on the basis of presence or absence of anticoagulation at admission, rather than using the proxy measure of prosthetic valves 3. These more recent studies not only show a low overall rate of intracerebral hemorrhage (2%), but also no increase in hemorrhage rates with anticoagulation (warfarin) therapy. Furthermore, they have reported a lower incidence of ischemic stroke in the patients taking anticoagulation at admission 4, 5.

In summary, there is a lack of evidence on which to base a decision regarding anticoagulation in endocarditis and stroke, because there are no randomized controlled trials in this area. Primum non nocere might come to mind, but interestingly, the very limited evidence in this area does not really support the idea that anticoagulation is harmful for patients such as ours. Indeed, one could argue there could be a hint of a possible benefit. Therefore, more studies of larger proportion, especially randomized trials should be undertaken in order to clarify this debatable question.

We accept that most clinicians would not anticoagulate without good evidence of benefit, but the decision is one for individual physicians considering individual patients.

## Learning Points/Take Home Messages

1. Infective endocarditis is a not a rare diagnosis, but one that can be missed. It is important that clinicians consider this condition, since it has a high mortality.
2. Stroke is a common embolic complication of infective endocarditis.
3. The most important treatment to prevent stroke in endocarditis is the initiation of antibiotic therapy.
4. It is unclear whether the initiation of de novo anticoagulation in patients with infective endocarditis is beneficial, since there are no randomized controlled trials in this area.
5. The only current available studies are of small sample size. However, the limited evidence does not suggest that anticoagulation causes harm in patients such as ours.
6. More studies, especially randomized trials in a larger population should be undertaken to study the effect of anticoagulation in patients with endocarditis presenting with thrombo‐embolic stroke.

## Conflict of Interest

None declared.
